# Travelling waves in a neural field model with refractoriness

**DOI:** 10.1007/s00285-013-0670-x

**Published:** 2013-04-02

**Authors:** Hil G. E. Meijer, Stephen Coombes

**Affiliations:** 1 Department of Applied Mathematics, MIRA Institute for Biomedical Engineering and Technical Medicine, University of Twente, Postbus 217, 7500 AE Enschede, The Netherlands; 2 Center for Mathematical Medicine and Biology, School of Mathematical Sciences, University of Nottingham, Nottingham, NG7 2RD UK

**Keywords:** Neural field models, Travelling waves, Refractoriness, Delay differential equations, 45J05 (37M20, 35C07)

## Abstract

At one level of abstraction neural tissue can be regarded as a medium for turning local synaptic activity into output signals that propagate over large distances via axons to generate further synaptic activity that can cause reverberant activity in networks that possess a mixture of excitatory and inhibitory connections. This output is often taken to be a firing rate, and the mathematical form for the evolution equation of activity depends upon a spatial convolution of this rate with a fixed anatomical connectivity pattern. Such formulations often neglect the metabolic processes that would ultimately limit synaptic activity. Here we reinstate such a process, in the spirit of an original prescription by Wilson and Cowan (Biophys J 12:1–24, 1972), using a term that multiplies the usual spatial convolution with a moving time average of local activity over some *refractory* time-scale. This modulation can substantially affect network behaviour, and in particular give rise to periodic travelling waves in a purely excitatory network (with exponentially decaying anatomical connectivity), which in the absence of refractoriness would only support travelling fronts. We construct these solutions numerically as stationary periodic solutions in a co-moving frame (of both an equivalent delay differential model as well as the original delay integro-differential model). Continuation methods are used to obtain the dispersion curve for periodic travelling waves (speed as a function of period), and found to be reminiscent of those for spatially extended models of excitable tissue. A kinematic analysis (based on the dispersion curve) predicts the onset of wave instabilities, which are confirmed numerically.

**Electronic supplementary material:**

The online version of this article (doi:10.1007/s00285-013-0670-x) contains supplementary material, which is available to authorized users.

## Introduction

The *continuum* approximation of neural activity can be traced back to work of Beurle (1956), who built a model describing the proportion of active neurons per unit time in a given volume of randomly connected nervous tissue. A major limitation of this very early neural field model is its neglect of refractoriness or any process to mimic the metabolic restrictions placed on maintaining repetitive activity. It was Wilson and Cowan (1972, 1973) who first developed neural field models with some notion of refractoriness. At the same time they also emphasised the importance of modelling neural population in terms of an excitatory subpopulation and an inhibitory subpopulation. Indeed over the years many studies of the Wilson-Cowan excitatory-inhibitory model have been made, with applications to problems in neuroscience ranging from the generation of electroencephalogram rhythms through to visual hallucinations, and see (Coombes et al. 2003) for a review. However, many of these subsequent studies drop the refractory term and focus more on the role of excitatory-inhibitory interactions in generating neural dynamics. Perhaps one exception to this is the work of Curtu and Ermentrout (2001), who have shown that the original Wilson-Cowan model with refractoriness can drive oscillations even in the absence of inhibition. Their work was done for a point model which begs the question as to whether refractoriness alone can allow for periodic waves to be generated in a spatially extended excitatory network. This is an especially intriguing issue given that neural field models with some form of inhibition or negative feedback, such as spike frequency adaptation, have traditionally been invoked to explain wave behaviour in cortex, including fronts, pulses, target waves and spirals (Ermentrout and McLeod 1993; Pinto and Ermentrout 2001; Huang et al. 2004).

In this paper we reinstate the original refractory term of Wilson and Cowan in a minimal neural field model describing a single population in one spatial dimension. This model is briefly reviewed in Sect. 2. In Sect. 3 we present a linear stability analysis, as well as a weakly nonlinear analysis, of the homogeneous steady state that predicts the onset of periodic travelling wave patterns in a purely excitatory network. This is confirmed by direct numerical simulations that show periodic travelling waves with profiles that appear as either single or multiple spikes of activity. A novel numerical continuation scheme is developed to track solution properties in a co-moving frame (speed, period, and profile shape) as a function of physiologically important system parameters (such as refractory time-scale, strength of anatomical connectivity, and firing threshold). These are obtained after recognising that the original model can be reformulated as a delay-differential equation for an exponentially decaying choice of anatomical weight distribution. The delay is set by the time-scale of the refractory process. In Sect. 4 we numerically construct the wave speed as a function of the wave period, to obtain the so-called dispersion curve. Here we avoid special case choices of the weight distribution and develop a numerical scheme that can handle the original delayed integro-differential model. The dispersion curve for an excitatory network with an exponentially decaying weight distribution is shown to have a shape reminiscent of that seen in the study of nonlinear reaction-diffusion systems, and in particular those arising in the study of an axon or active dendrite (Miller and Rinzel 1981). Using the dispersion curve we further develop a kinematic model that allows predictions about non-regular spike trains to be made, including period-doubling scenarios subsequently confirmed by direct numerical simulations. In addition, we establish an example of a homoclinic orbit of chaotic saddle-focus type in an infinite-dimensional system. Finally in Sect. 5 we present a brief discussion of the work in this paper.

## The Wilson-Cowan model with refractoriness

Wilson and Cowan considered the spatio-temporal evolution of the activity of synaptically interacting excitatory and inhibitory neural sub-populations (Wilson and Cowan 1972). A recent review of their model can be found in (Coombes et al. 2013; Bressloff 2012). A common reduction of their original model, and one often employed as a minimal model of cortex, takes the form of a scalar integro-differential equation:

1 $$\begin{aligned} \tau \frac{\partial u}{\partial t} = -u + f(w \otimes u) . \end{aligned}$$

Here $u=u(x,t) \in {\mathbb{R }}$ is a temporal coarse-grained variable describing the proportion of neural cells firing per unit time at position $x \in {\mathbb{R }}$ at the instant $t\in {\mathbb{R }}^+$. The symbol $\otimes$ represents spatial convolution, the function $w$ describes an effective anatomical connectivity or weight distribution and is a function of the distance between two points, and $\tau$ is the relaxation time-scale. The nonlinear function $f$ describes the expected proportion of neurons receiving at least threshold excitation per unit time, and is often taken to have a sigmoidal form. In major contrast to the original Wilson-Cowan equations refractory terms are not included in this model. To reinstate such terms in (1) we follow (Wilson and Cowan 1972), and more recently (Curtu and Ermentrout 2001), and model the fraction of cells in their absolute refractory period $R$ by

2 $$\begin{aligned} z(x,t)=\frac{1}{R}\int _{t-R}^{t}u(x,s ){\text{ d }}s. \end{aligned}$$

Since only a fraction $(1-z)$ of cells can be activated and actually contribute to any firing activity the model (1) is modified to

3 $$\begin{aligned} \tau \frac{\partial u}{\partial t} = -u + (1-z)f(w \otimes u). \end{aligned}$$

For convenience we rescale time $t\mapsto Rt$ and define $r=R/\tau$ to obtain the model that we shall work with for the remainder of this paper:

4 $$\begin{aligned} \frac{1}{r}u_{t}=-u+\left( 1-\int _{t-1}^{t}u(x,s){\text{ d }}s\right) f(w\otimes u). \end{aligned}$$

As a choice of firing rate we shall take the sigmoid

5 $$\begin{aligned} f(u)=\frac{1}{1+{\text{ e }}^{-\beta (u-\theta )}}, \end{aligned}$$

with threshold $\theta$ and steepness parameter $\beta$. For the choice of weight distribution we shall consider symmetric normalised kernels such that $w(x)=w(|x|)$ and $\int _{-\infty }^\infty w(y) {\text{ d }} y =1$.

## Analysis of waves

Direct numerical simulations of a purely excitatory network, see below, show the possibility of periodic travelling waves. This is particularly interesting because these are not typically found in neural field models with pure excitation, though they are often encountered in the presence of some form of negative feedback, such as may arise with the inclusion of an inhibitory sub-population or a form of spike frequency adaptation, as reviewed in (Ermentrout 1998). If these patterns arise via the instability of the homogeneous steady state, then they can be predicted using a classic Turing instability analysis. Their analysis beyond the point of instability can be pursued with a weakly nonlinear analysis, to develop a set of amplitude equations (typically in the form of coupled complex Ginzburg-Landau equations), as in (Curtu and Ermentrout 2004; Venkov et al. 2007). However, this is only relevant close to the bifurcation point, and it is much more informative to gain an insight into the fully nonlinear properties of waves using numerical analysis. This has been pursued at length for many excitable systems, and especially for single neuron models of the axon or active dendrite with single (Miller and Rinzel 1981; Röder et al. 2007) or multi-pulse (Evans et al. 1982; Feroe 1982; Hastings 1982; Kuznetsov 1994; Lord and Coombes 2002) periodic waves. However, the study of periodic travelling waves has largely been ignored in the neural field community, which is surprising since this can inform a kinematic analysis [elegantly reviewed in (Keener and Sneyd 1998)] to predict instabilities to more exotic classes of travelling wave solution. We build on a Turing analysis and develop precisely this approach below.

### Linear stability analysis of homogeneous solutions

A homogeneous fixed point with $u(x,t)={\overline{u}}$ is given by the solution of the nonlinear algebraic equation

6 $$\begin{aligned} \frac{{\overline{u}}}{1-{\overline{u}}} = f({\overline{u}}). \end{aligned}$$

**Fig. 1:**
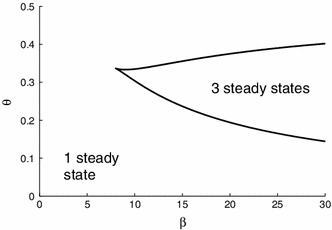
The boundaries in the $(\beta ,\theta )$-plane with 3 fixed points

The model displays up to three different fixed points depending on $\beta$ and $\theta$, see Fig. 1, which we denote by $u_{i}$ with $u_{1}<u_{2}<u_{3}$ when three fixed points exist.

Linearising around ${\overline{u}}$ and considering perturbations of the form ${\text{ e }}^{ikx} {\text{ e }}^{\lambda t}$ gives a dispersion relation for the pair $(\lambda ,k)$ in the form $\mathcal{E }(\lambda ,k)=0$, where

7 $$\begin{aligned} \mathcal{E }(\lambda ,k)&= 1+\frac{\lambda }{r} + f({\overline{u}}) \frac{1}{\lambda }(1-{\text{ e }}^{-\lambda }) - (1-{\overline{u}}) f^{\prime }({\overline{u}}) {\widehat{W}}(k), \end{aligned}$$

8 $$\begin{aligned} {\widehat{W}}(k)&= \int _{-\infty }^\infty w(y) {\text{ e }}^{iky} {\text{ d }} y. \end{aligned}$$

The Turing bifurcation point is defined by the smallest non-zero wave number $k_c$ that satisfies $\text{ Re }~ (\lambda (k_c)) =0$. It is said to be *static* if $\text{ Im }~ (\lambda (k_c)) =0$ and *dynamic* if $\text{ Im }~ (\lambda (k_c)) \equiv \omega _{c} \ne 0$. A static bifurcation may then be identified with the tangential intersection of $\omega =\omega (\nu )$ and $\nu =0$ at $\omega =0$. Similarly a dynamic bifurcation is identified with a tangential intersection with $\omega \ne 0$. Beyond a dynamic instability one would expect the emergence of a periodic travelling wave of the form ${\text{ e }}^{i(k_c x +\omega _c t)}$ (with speed $\omega _c/k_c$).

For the sigmoidal function (5) we have that $f^{\prime }=\beta f (1-f)$. For an exponential kernel $w(x)=S {\text{ e }}^{-S|x|}/2$, with $S>0$, then ${\widehat{W}}(k) = (1+(k/S)^2)^{-1}$, which has a maximum of one at the origin. Hence for $\lambda \in {\mathbb{R }}$ we have that $\lim _{\lambda \rightarrow 0}\mathcal{E }(\lambda ,k)=-A(k)+f({\overline{u}})$ where

9 $$\begin{aligned} A(k)=-1+(1-{\overline{u}}) f^{\prime }({\overline{u}}) {\widehat{W}}(k)=-1+\beta \frac{{\overline{u}}(1-2{\overline{u}})}{1-{\overline{u}}}{\widehat{W}}(k). \end{aligned}$$

For large $r$ we see that $\mathcal{E }(\lambda ,0)$ is a decreasing function of $\lambda$ so that a fixed point will be stable (to a static instability with $k_c=0$) if $\lim _{\lambda \rightarrow 0}\mathcal{E }(\lambda ,0) <0$, or equivalently, $\beta >\beta _\text{ s }$ where

10 $$\begin{aligned} \beta _\text{ s } = \frac{1}{{\overline{u}}(1-2 {\overline{u}})}. \end{aligned}$$

For $\lambda \in {\mathbb{C }}$ it is natural to write $\lambda = \nu + i \omega$ and equate real and imaginary parts of (7) to obtain two equations for $\nu$ and $\omega$, which we write in the form

11 $$\begin{aligned} G(\nu ,\omega ) \equiv \text{ Re }~ \mathcal{E }(\lambda ,k)=0 , \quad H(\nu ,\omega ) \equiv \text{ Im }~ \mathcal{E }(\lambda ,k) =0 . \end{aligned}$$

The simultaneous solution of these two equations gives the pair $(\nu (k),\omega (k))$. For $\lambda =i \omega$ the above pair of equations reduces to

12 $$\begin{aligned} A(k) =\frac{f({\overline{u}})\sin \omega }{\omega } , \quad f({\overline{u}}) r = \frac{\omega ^2}{1-\cos (\omega )}. \end{aligned}$$

Since there are singularities at $\omega = 2 n \pi$, these equations define a series of parametric curves $T_i=T({\overline{u}}_i)$ defined in the regions $\omega \in (2n \pi , 2(n + 1)\pi )$ for $n \in {\mathbb{Z }}$. We see that there will be a solution if and only if

13 $$\begin{aligned} rA(k) = \frac{\omega \sin \omega }{1-\cos \omega }. \end{aligned}$$

**Fig. 2:**
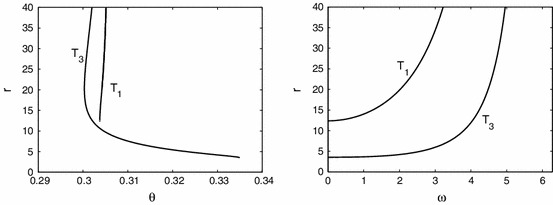
The dependence of the Turing curves $T$ on $r$ and $\theta$ with corresponding $\omega$. The $T_{1}$ curve corresponds to the lower fixed point, $T_{3}$ corresponds to the higher fixed point. Other parameters are $k=2\pi /10, S=10, \beta =10$

It is clear that (13) defines a quadratic function in ${\overline{u}}$ and it turns out that $T_{2}$ does not exist, only $T_{1}$ and $T_{3}$. In Fig.  2 we show only the branches with $\omega <2\pi$ as we did not find any with larger $\omega$. Using the observation that $\sin (\omega )/\omega <1$ and $(1-\cos \omega )/\omega ^2 <1/2$ we see that a solution for $\omega _c \ne 0$ is only possible if $f({{\overline{u}}})>A(0)$ and $r>2/f({\overline{u}})$. Hence a dynamic instability will occur before a static instability when $\beta <\beta _\text{ s }$ and $r$ is sufficiently large. To determine whether the static instability gives rise to a travelling or a standing wave it is useful to perform a weakly nonlinear analysis.

### Weakly nonlinear analysis: amplitude equations

A characteristic feature of the dynamics of systems beyond an instability is the slow growth of the dominant eigenmode, giving rise to the notion of a *separation of scales*. This observation is key in deriving the so-called *amplitude equations*. In this approach information about the short-term behaviour of the system is discarded in favour of a description on some appropriately identified slow time-scale. By Taylor-expansion of the dispersion curve near its maximum one expects the scalings $\text{ Re } \, \lambda \sim r-r_c, \ k-k_c \sim \sqrt{r-r_c}$, close to bifurcation, where $r$ is the bifurcation parameter. Since the eigenvectors at the point of instability are of the type $Z_L {\text{ e }}^{i(\omega _c t +k_c x)} + Z_R {\text{ e }}^{i(\omega _c t -k_c x)} + \text{ cc }$, for $r>r_c$ emergent patterns are described by an infinite sum of unstable modes (in a continuous band) of the form ${\text{ e }}^{\nu _0(r-r_c) t} {\text{ e }}^{i(\omega _c t +k_c x)} {\text{ e }}^{i k_0\sqrt{r-r_c} x}$. Let us denote $r=r_c+\epsilon ^2\delta$ where $\epsilon$ is arbitrary and $\delta$ is a measure of the distance from the bifurcation point. Then, for small $\epsilon$ we can separate the dynamics into fast eigen-oscillations ${\text{ e }}^{i(\omega _c t +k_c x)}$, and slow modulations of the form ${\text{ e }}^{\nu _0\epsilon ^2 t}{\text{ e }}^{i k_0\epsilon x}$. If we set as further independent variables $T = \epsilon ^2 t$ for the modulation time-scale and $X = \epsilon x$ for the long-wavelength spatial scale (at which the interactions between excited nearby modes become important) we may write the weakly nonlinear solution as $Z_L(X,T) {\text{ e }}^{i(\omega _c t +k_c x)} + Z_R(X,T) {\text{ e }}^{i(\omega _c t -k_c x)} +\text{ cc }$. It is known from the standard theory (Hoyle 2006) that weakly nonlinear solutions will exist in the form of either travelling waves (TWs), $Z_L = 0$ or $Z_R = 0$, or standing waves (SWs), $Z_L = Z_R$. In the Appendix we show that (ignoring spatial variation) the amplitude equations take the form

14 $$\begin{aligned} \frac{\text{ d } Z_L}{\text{ d } T}&= [{\varLambda } + {\varPhi } |Z_L|^2 + {\varPsi } |Z_R|^2]Z_L, \end{aligned}$$

15 $$\begin{aligned} \frac{\text{ d } Z_R}{\text{ d } T}&= [{\varLambda } + {\varPhi } |Z_R|^2 + {\varPsi } |Z_L|^2]Z_R , \end{aligned}$$

where ${\varLambda },\, {\varPhi }$ and ${\varPsi }$ are known functions of system parameters. A linear stability analysis of the above amplitude equations generates the conditions for selection between TWs or SWs. If $\text{ Re }\, {\varLambda }$ and $\text{ Re }\, {\varPhi }$ have opposite sign, then a TW exists and for $\text{ Re }\,{\varPhi }<0$ and $\text{ Re }\, ({\varPhi } -{\varPsi }) >0$ it is stable. If $\text{ Re }\, {\varLambda }$ and $\text{ Re }\, ({\varPhi } +{\varPsi })$ have opposite sign, then a SW exists and for $\text{ Re }\, ({\varPhi } +{\varPsi })<0$ and $\text{ Re }\, ({\varPhi } - {\varPsi }) <0$ it is stable. Evaluation of the coefficients ${\varPhi }$ and ${\varPsi }$ (see Appendix), yields $\text{ Re }\, {\varPhi } >0$ and $\text{ Re }\, ({\varPhi } +{\varPsi })>0$. Therefore, for typical parameter values the Turing instability is subcritical and travelling and standing waves are unstable. Also, along $T_{3}$, waves exist for $\delta >0$ if $r>r_{s}\approx 20.3$ and for $\delta <0$ if $r<r_{s}$. Still, these can be used to start to track waves numerically. Note that a similar weakly nonlinear analysis for waves in a neural field model without refractoriness though with adaptation has been performed in (Curtu and Ermentrout 2004), and for axonal delays in (Venkov et al. 2007).

In the next part we will consider waves and how these can grow beyond a dynamic Turing bifurcation.

### Excitability and waves

From the Turing analysis above we expect to see travelling waves for sufficiently large $r$ and suitable $\theta$. A similar observation, based on the numerical simulation of a lattice model with nearest-neighbour coupling has previously been made by Curtu and Ermentrout (2001). These authors further point out that for some range of $\beta$ values that the point version of the model (obtained with the choice $w(x)=\delta (x)$) can be viewed as an excitable system. The excitability is easily recognised if we consider the spatially homogeneous system $\dot{u}=r[-u +(1-z)f(u)]$, with $u=u(t)$ and $z=\int _{t-1}^t u(s) {\text{ d }} s$. This can be re-written in delay-differential form as

16 $$\begin{aligned} \dot{u}=r\left( -u+(1-z)f(u) \right) ,\quad \dot{z}=u(t)-u(t-1). \end{aligned}$$

**Fig. 3:**
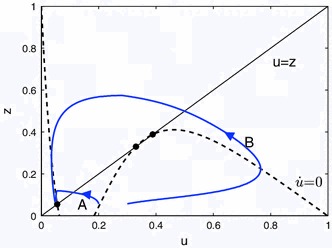
The *dashed line* indicates the $u$-nullcline $z=1-u/f(u)$ with $\beta =10,\theta =0.333$. The steady condition $u=z$ (*solid black line*) intersects the nullcline at 3 points indicated by *circles*. The trajectories (*blue*) start from (*A*) $u(0)=0.2$ and (*B*) $u(0)=0.3$ and history $u(\tau )=0.058$ for $-1<\tau < 0$, i.e. they have been given an initial kick. They approach the lower steady state at ${\bar{u}}\approx 0.0554$ (colour figure online)

We graph the nullcline of the $u$-equation $z=1-u/f(u)$ together with the steady states constraint $u=z$ and two trajectories in Fig. 3. These show that if an initial displacement from the steady state is sufficiently large, then the trajectory makes a large excursion. In other words, the system is excitable.

**Fig. 4:**
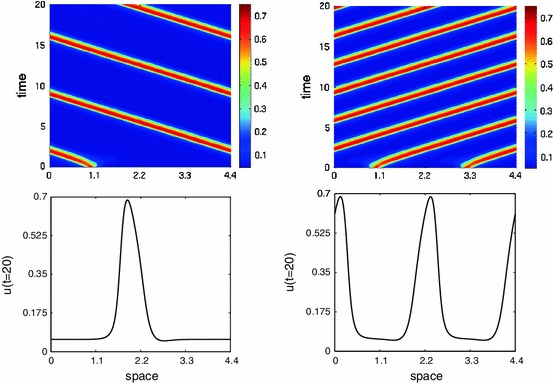
*Left* a left travelling wave for (4). *Right* a right travelling 2 pulse wave. The patterns seem to settle after a transient time around $t=5$. The lower figures show the profile of the solution $u$ at $t=20$. Parameters are $S=10,\beta =10,\theta =.333,r=10$ (colour figure online)

Next we want to show travelling waves for the full spatially extended system defined by (4) using direct numerical simulations. We evolve the state as follows. We use an equidistant spatial discretisation with $2^{11}$ mesh points with periodic boundary conditions. We compute the spatial convolution using Fourier transforms. The history integral $z(x,t)=\int _{t-1}^{t}u(x,s) {\text{ d }} s$ is calculated using a trapezoidal rule with $100$ points. This gives a system that can be simulated with matlab’s dde23-solver. Supplying the history as $u(x,\tau )=.05+0.7\exp (-80(x-1-0.63\tau )^2)$ we obtain a travelling wave, see Fig. 4 (left). Other initial history can also lead to travelling waves as long as the amplitude at one spot is sufficient to cause excitation of neighbouring tissue and the initial spot decays due to refractoriness. The figure suggests that there is enough space to fit in a second moving pulse and this is indeed possible, see Fig. 4 (right). Note that the time from the one pulse to the next is different than from the previous pulse. The travelling waves shown in Fig. 4 have nearly the same velocities namely $c\approx 0.6302$ for the travelling one pulse, and $c\approx 0.6309$ for the two pulses. The trajectories near the steady state in Fig. 3 also go some way to explaining why the two pulses can move slightly faster. For two pulses in one periodic domain the next pulse arrives when the system is less refractory, i.e. $z$ is lower, than with only one pulse. We study the dependence of the speed on the wavenumber below.

If the domain for the wave becomes infinite, the travelling wave approaches a pulse which is a homoclinic orbit. The linear stability of the steady state in the moving frame $\xi =x+ct$ classifies the homoclinic orbit. All travelling waves profiles can be constructed as stationary profiles of (4) in the travelling frame $\xi =x+ct$, namely as solutions of the dynamical system:

17 $$\begin{aligned} \frac{c}{r}u_{\xi }=-u+\left( 1-\frac{1}{c}\int _{\xi -c}^{\xi } u(s) {\text{ d }} s\right) f \left( \;\int _{{\mathbb{R }}}w(y)u(\xi -y) {\text{ d }} y\right) . \end{aligned}$$

Focusing now on the homoclinic orbit we consider small perturbations $u(\xi )={\bar{u}}+v(\xi )$ to obtain

18 $$\begin{aligned} \frac{c}{r}v_{\xi }=-v+(1-{\bar{u}})f^{\prime }(w\otimes {\bar{u}})\int _{{\mathbb{R }}}w(\xi )v(\xi -y) {\text{ d }} y -f(w\otimes {\bar{u}})\frac{1}{c}\int _{\xi -c}^{\xi }v(s) {\text{ d }} s .\qquad \end{aligned}$$

This has solutions of the form $v=\exp (\lambda \xi )$ where $\lambda$ is a solution of the transcendental equation

19 $$\begin{aligned} -c\lambda /r-1+(1-{\bar{u}})f^{\prime }({\bar{u}})\frac{S^{2}}{S^{2}-\lambda ^2} -f({\bar{u}})\frac{1}{c\lambda }\left( 1-{\text{ e }}^{-c\lambda }\right) =0. \end{aligned}$$

**Fig. 5:**
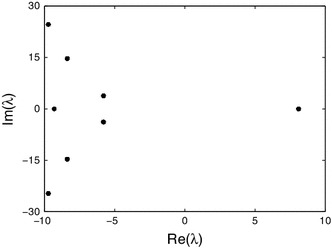
The eigenvalues of the fixed point ${\bar{u}}=.05537499$ for $c=0.6303,\, S=10,\, \beta =10,\, \theta =.333$, and $r=10$. Thus the homoclinic orbit can be classified as one of saddle-focus type with saddle-quantity $>1$

Here we impose the condition $|\text{ Re }\,(\lambda )|<S$ to ensure convergence of the integral over ${\mathbb{R }}$ in (18). Fortunately, this includes the imaginary axis allowing stability analysis. If $S\rightarrow \infty$ we recover the formula derived in (Curtu and Ermentrout 2001) for the Hopf bifurcation of the point model. Solving for the (single) steady state we find ${\bar{u}}\approx 0.0554$. We insert the wavespeed $c=0.6303$ as observed in the simulations and numerically solve the eigenvalue equation (19) for many random starting values near the origin in the complex plane, see Fig. 5. We find a single positive real eigenvalue $\lambda _1\approx 8.1902$ and the other eigenvalues are complex pairs with negative real part. The leading stable eigenvalues are $\lambda _{2,3}\approx -5.8021 \pm 3.8026i$ (and satisfy the constraint $|\text{ Re }\,(\lambda )|<S$). We conclude that ${\bar{u}}$ is a saddle-focus with saddle-quantity $\lambda _{1}/\text{ Re } \,(\lambda _{2,3})>1$. This implies the existence of N-homoclinic loops for all $N=1,2,3, \ldots$. In particular, we may expect that travelling waves with 3 pulses form an isolated branch and have a saddle-node bifurcation (Gonchenko et al. 1997), even though the system is infinite-dimensional. It is an open challenge to rigorously prove this (and extend the result from the finite dimensional setting). However, it is likely that Lin’s method can be applied in this case, along the lines considered in (Lin 1990) for analysing the bifurcation of a unique periodic orbit from a homoclinic orbit to an equilibrium in a DDE.

### Numerical continuation of waves

Here we start with the derivation of an equivalent DDE in a co-moving frame for the travelling waves. This is a standard approach and normally would allow us to study periodic orbits that relate to waves in the original system. However, we found that for the available numerical tools to work we needed to modify the equations artificially. Therefore we computed the waves in an alternative and novel way. Rather than using a PDE approach we inserted the co-moving frame directly and used the discretisation of that system as described below.

#### A delay differential equation for waves

We write $z(x,t)=\int _{t-1}^{t}u(x,s) {\text{ d }} s$ and transform (4) to a delayed partial differential equation (DPDE) using Fourier techniques (see (Coombes et al. 2003) for more details) to obtain

20 $$\begin{aligned} \left( 1+\frac{1}{r}\partial _{t}\right) u&= (1-z)f(\psi ), \nonumber \\ \partial _{t}z&= u(x,t)-u(x,t-1), \nonumber \\ \left( S^{2}-\partial _{x}^{2}\right) \psi&= S^{2}u. \end{aligned}$$

Next we insert the wave ansatz $\xi =t+x/c$ and obtain the following delay differential equation (DDE)

21 $$\begin{aligned} u_\xi&= r\left( -u+(1-z)f(\psi )\right) ,\nonumber \\ z_\xi&= u(\xi )-u(\xi -1),\nonumber \\ \psi _\xi&= \phi ,\nonumber \\ \phi _\xi&= -(cS)^{2}\left( u-\psi \right) . \end{aligned}$$

One can make the following observations about this equivalent DDE. First, suppose we had inserted the more common ansatz $\xi =x-ct$ for right-going waves. We would then have obtained an advanced instead of a delayed term. Also, this time rescaling gives a constant delay which is numerically more stable. Second, for simulations one can also compute the history integral from the DDE for $z$ in (21). However, the history of $z$, must satisfy the constraint

22 $$\begin{aligned} z(\tau )=\int _{\tau -1}^{\tau }u(s) {\text{ d }} s, \quad \text{ for } \tau \in (-1,0]. \end{aligned}$$

So, if we know the history of $z$ we actually know $u(\tau )$ for $\tau \in (-2,0]$. We use system (21) to determine periodic orbits with numerical continuation. The continuation problem automatically specifies enough history so that this does not pose a problem. Solving (21) using knut (Roose and Szalai 2007) we did not achieve convergence. This can be understood from the steady state problem of system (16). This is ill-defined as any constant may be added to $z$ giving a continuum of solutions as we lose the constant of integration. The integral constraint yields $z={\bar{u}}$ and hence maximally three steady states exist. Adding a small additional term $\varepsilon (u-z)$ to $z_\xi$ in (16) and (21) with $\varepsilon =10^{-6}$ incorporates the integral constraint. The continuation results for both system (16) and (21) were then numerically stable and in agreement with final profiles obtained from simulations.

#### Direct continuation of the integral equation

As the DDE-approach only works for the modified system, we develop a novel numerical scheme to track periodic solutions of the integral equation in a co-moving frame. The novelty is that we do not introduce auxilary variables as for the DDE, but compute the (convolution) integrals directly using fast Fourier transforms (FFT). Working with the non-local model (17) directly also allows us to treat a more general class of weight distributions and not only those of exponential form (though we do not pursue this here).

Similarly as for the simulations of (4) we use an equidistant spatial grid $\xi$ with $N$ mesh points for the interval $[0,{\varDelta }]$. We employ central finite differences for the temporal derivative. We have one convolution with the connectivity $w$. For our periodic solutions $u$, this convolution can be computed by taking the FFT of $w$ and $u$, multiplying element-wise and then applying the inverse FFT. It is sufficient to take the FFT of $w$ and not its periodic summation as the connectivity $w$ decays sufficiently fast so that $w(-{\varDelta }/2)\approx 0$. Hence the circular convolution theorem can be employed. We observe that the integral over $[\xi -c,\xi ]$ can be seen as another convolution of $u$ with $g=H(\xi -c)H(-\xi )$ with $H$ the Heaviside step function. It is not strictly necessary, but we have the Fourier transforms of $g$ and $w$ analytically and can evaluate them immediately without transforming these with a FFT. For simplicity and convergence, we use $N=2^{13}$. Next we use the periodic boundary condition $u_{0}=u_{N+1}$ to eliminate one equation. Then to break the translational invariance we add the integral phase condition $\int (u-u_0)\dot{u} {\text{ d }} \xi =0$ where $u_0$ is some reference solution; here we take the previously computed point. This results in $N+1$ equations for $N+1$ unknowns. Then we use the pseudo-arclength condition $\langle v,(x-x_0)\rangle =h$, where $v$ is the tangent vector, $h$ is the step-size and the brackets indicate the standard inner-product (Meijer et al. 2009). We add the spatial period ${\varDelta }$ as an additional parameter.

#### Initial data for the continuation

The numerical continuation of travelling waves needs an initial point sufficiently close to an actual solution branch. There are two ways to obtain such data. The first is to take parameters corresponding to a Turing instability. The initial profile is then $u(\xi )={\bar{u}}+\varepsilon \cos (\xi )$. The second way is to use a simulation where $u$ approaches a travelling wave. Then the final profile $u_{p}$ of $u$ can be used as initial point for the continuation. This is sufficient for our novel method. For the DDE, we need the auxilary variables $z,\psi ,\phi$ along the periodic orbit. For $z$ we integrated $u_{p}$ with the trapezoidal rule over $[\xi -c,\xi ]$. Convoluting $u_{p}$ with the connectivity $w$ yields $\psi$ and $\phi$ is obtained from numerical differentation of $\psi$. The initial parameters are the same as in the simulation.

#### Parameter dependence of the travelling waves

**Fig. 6:**
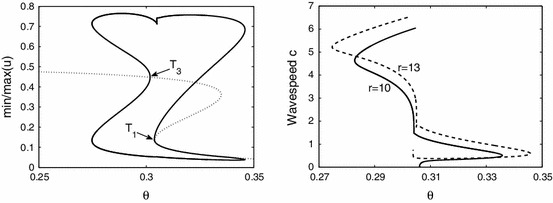
*Left* The minima and maxima of the wave profile for varying $\theta$ for $r=13$ (*solid line*). The wave emanates from the Turing points at $T_{1,3}$ from the homogeneous fixed point (*dotted line*). *Right* The dependence of the wavespeed when varying the threshold $\theta$ for $r=10$ (*solid*) and $r=13$ (*dashed*). The waves with maximal amplitude are stable, the others are unstable. Spatial domain always fixed to ${\varDelta }=10$ and $S=\beta =10$

**Fig. 7:**
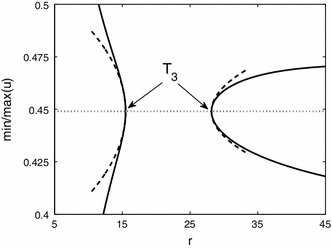
The minima and maxima of the wave profile varying $r$ for $\theta =0.3008$ (*solid line*). The travelling wave emanates from Turing points at $T_{3}$ for $\theta =0.3008$ from the steady state (*dotted line*). The amplitude predicted by the weakly nonlinear analysis is indicated by *dashed lines*. Spatial domain always fixed to ${\varDelta }=10$ and $S=\beta =10$

**Fig. 8:**
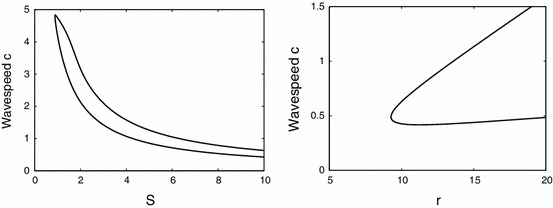
The dependence of the wavespeed for the 1 pulse solution when varying connectivity scale $S$ (*left*) and refractory time $r$ (*right*). The upper (*lower*) branch corresponds to stable (unstable) solutions. Spatial domain always fixed to ${\varDelta }=10$

From Fig. 2 we find Turing instabilities for $r=13$ at $\theta _{1}=0.3038$ and $\theta _{3}=0.3018$ with $\omega =0.6229$ and $\omega =4.088$, respectively. Starting the numerical continuation from $T_{3}$ and varying $\theta$ we find travelling waves, see Fig. 6. First they are unstable, but the branch turns at $\theta =0.2747$ where the travelling waves are stable until $\theta =0.3458$. In between, near $\theta =0.305$, there is bistability of two different waves where the branch turns twice. The difference in the profile is an additional local minimum, present for lower values of $\theta$. Finally, the branch ends at $T_{1}$. For $r=10$ there is only one Turing instability for $\theta =0.3046$ with $\omega =3.7941$. Following this branch we encounter similar scenarios, but the branch ends by approaching a non-uniform stationary profile near $\theta =0.3060$ when the speed $c$ vanishes. Also the amplitude shows that the wave emanates from these Turing points (at $T_{1,3}$). The amplitude of travelling waves near Turing points is also illustrated in Fig. 7. This shows for fixed $\theta =0.3008$ that the prediction of the amplitude equations and results of the numerical continuation match well. We were unable to observe these small amplitude waves in simulations close to the Turing points corroborating that these bifurcations are subcritical. We have also investigated the effect of other system parameters, see Fig.  8. For decreasing $S$ the wave speed increases as waves more easily excite neighbouring tissue. Increasing $r$ leads to faster stable waves.

## Dispersion curves and kinematic theory

**Fig. 9:**
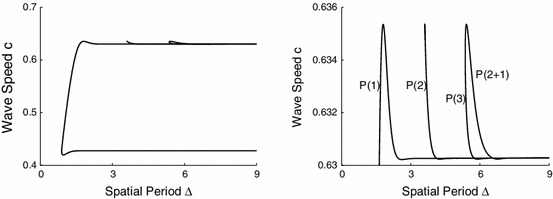
Dispersion curves of travelling waves with N=1–3 pulses. *Left* enlargement near the upper branch. The *symbols* $P(n)$ indicate the number of pulses within a period ${\varDelta }$ and $P(2+1)$ differs from $P(3)$ in that the interpulse distance is different, see Fig. 11. The $P(1)$ solution is expected to be stable (using a kinematic analysis) when $c^{\prime }({\varDelta })>0$

Figure 9 shows the dependence of the wave speed on the spatial period ${\varDelta }$. The direct continuation of the integral equation and those obtained using (21) agreed very well, i.e. to the first four digits.

A *kinematic* theory of wave propagation is one attempt to follow the progress of localised pulse shapes, within a periodic wave, at the expense of a detailed description of their shape (Rinzel and Maginu 1984). Suppose that a pulse has a well defined arrival time at some position $x$ then we denote the arrival of the $n$th pulse at position $x$ by $T^n(x)$. A periodic wave, of period ${\varDelta }$, is then completely specified by the set of ordinary differential equations

23 $$\begin{aligned} \frac{\text{ d } T^n(x)}{\text{ d } x} = \frac{1}{c({\varDelta })}, \end{aligned}$$

with solution $T^n(x)=n {\varDelta } + x/c$, where $c=c({\varDelta })$ is the dispersion curve, such as that obtained numerically in Fig. 9. The kinematic formalism asserts that there is a description of irregular spike trains in the above form such that

24 $$\begin{aligned} \frac{\text{ d } T^n(x)}{\text{ d } x} = \frac{1}{c(T^{n}(x) - T^{n-1}(x))}, \end{aligned}$$

where $T^{n}(x) - T^{n-1}(x)$ is recognised as the instantaneous period of the wave train at position $x$. A steadily propagating wave train is stable if under the perturbation $T^n(x) \rightarrow T^n(x) + u^n(x)$ the system converges to the unperturbed solution during propagation, or $u^n(x) \rightarrow 0$ as $x\rightarrow \infty$. For the case of uniformly propagating periodic traveling waves of period ${\varDelta }$ we insert the perturbed solution in (23), so that to first order in the $u^n$

25 $$\begin{aligned} \frac{\text{ d } u^n}{\text{ d } x} = - \frac{c^{\prime }({\varDelta })}{c^2({\varDelta })} [u^n - u^{n-1}]. \end{aligned}$$

Thus, a uniformly spaced, infinite wave train with period ${\varDelta }$ is stable (within the kinematic approximation) if and only if $c^{\prime }({\varDelta }) >0$. Hence, for the dispersion curves of shown in Fig. 9 it would seem to a first approximation that it is always the faster of the two periodic branches that is stable. Note that where there are *bumps* in the dispersion curve defining so-called supernormal wave speeds (wave speeds are faster than the corresponding speed of the large period wave) then it is only the supernormal wave of smaller period that is stable. Corresponding conclusions can also be made about subnormal waves (waves of slower speed compared to the wave of large period) on the slower branch. Also shown in Fig. 9 are waves with multiple pulses per period ${\varDelta }$ and we indicate the number $n$ of pulses on a branch by $P(n)$.

**Fig. 10:**
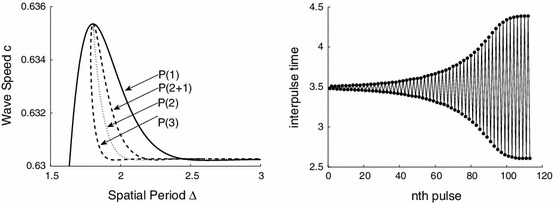
*Left* Scaled dispersion curves on the upper branch. *Right* The inter-pulse time for successive pulses for ${\varDelta }=4.4$ and two pulses in the domain. Initially equal as along the P(1) dispersion curve and then as along the P(2) branch. See the animation (Online Resource 1) for the simulation

According to the kinematic prediction there is a change of stability at the stationary points of the dispersion curve, i.e. the extrema in Fig. 9. As the branch persists another solution branch must bifurcate from a stationary point. Therefore we expect these points to act as organising centers of the waves. Indeed with (21) we have verified that the 2-pulse solution starts from a period-doubling bifurcation very close to the highest stationary point. In addition we plotted the dispersion curve with $({\varDelta }/N)$, i.e. period per pulse, see Fig. 10. This may be demonstrated on an infinite domain, but here we show the following simulations. Consider a periodic domain ${\varDelta }=4.4$, where the $P(2)$ dispersion curve has positive slope. If we consider two pulses at equal distance then this is equivalent to the $P(1)$ branch at ${\varDelta }=2.2$. Indeed, this is the result found in Fig. 4 (right), where we determined the wavespeed $c\approx 0.6310$. Now we take a slightly displaced double one-pulse solution, i.e. one pulse starts at $x=1$ and the other at $x=3.19$. Initially these pulses travel as two separate pulses but then adjust their speeds, and inter-pulse time, to travel together at a slightly lower speed $c=0.6302$, see Fig. 10.

**Fig. 11:**
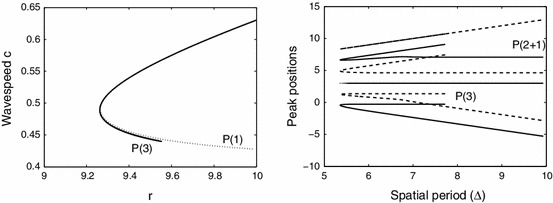
*Left* The 3 pulse solution forms an isolated branch in a two parameter diagram. *Right* The spatial positions of the peaks of the pulses on the three pulse branch. We have plotted two fundamental domains and centred the six pulses around the third pulse

The travelling wave solution with three pulses corroborated the chaotic saddle-focus scenario even further as it formed an isolated branch in a two parameter diagram as expected, Fig. 11 (left). Interestingly we found that the $P(3)$ and $P(2+1)$ branch were different in the inter-peak distance, see Fig. 11 (right). On the $P(3)$ branch the three pulses travel together where the distance between the first and second and that of the second and third is nearly equal around $1.639$, while on the $P(2+1)$ branch the third pulse follows at a distance of $2.45$ from the second.

## Discussion

We have considered periodic travelling waves in a one dimensional neural field model describing a single spatially extended population with purely excitatory interactions. Importantly we have included an absolute refractory process as in the original work of Wilson and Cowan (1972) and shown how to analyse this using a mixture of linear (Turing) analysis and novel numerical techniques. Despite the long history and extensive study of this type of model, to the best of our knowledge this is the first analysis of moving $N$-pulses in a neural field model with refractoriness. Moreover, we have shown that the types of travelling pulse patterns in this class of neural field model can be captured with a reduced kinematic description. This highlights the importance of the shape of the dispersion curve and its usefulness in predicting the behaviour of more exotic travelling wave packets. Given that other variants of neural field models, such as those that include axonal delays (Venkov et al. 2007), synaptic depression (Kilpatrick and Bressloff 2010), and slow inhibitory feedback (Taylor and Baier 2011) are also known to support periodic travelling waves it is of interest to construct dispersion curves for these models and contrast their shapes (and in effect the types of wave that they would be able to support). This is a topic of ongoing research and will be reported upon elsewhere.

### Electronic supplementary material

Below is the link to the electronic supplementary material.

Supplementary material 1 (mpg 8621 KB)

## Appendix

Let us consider the value of $r$ at the bifurcation point to be given by $r=r_c$ with corresponding values of $\omega$ and $k$ as $\omega _c$ and $k_c$ respectively. We consider an asymptotic expansion for $u$ in the form $u={\overline{u}}+ \epsilon u_1 +\epsilon ^2 u_2 +\epsilon ^3 u_3 + \ldots$, where $u_i=u_i(x,t,\epsilon ^2 t)$. After setting $T=\epsilon ^2 t$ and $r=r_c+\epsilon ^2 \delta$ we then substitute into (4), making use of the fact that $\partial _t \rightarrow \partial _t + \epsilon ^2 \partial _T$ and $u_i(x,s,\epsilon ^2 s) \approx u_i(x,s,T)+\epsilon ^2(s-t) \partial u_i(x,s,T)/\partial {T}$. Equating powers of $\epsilon$ leads to a hierarchy of equations:

26 $$\begin{aligned} {\overline{u}}&= (1-{\overline{u}})\beta _0, \end{aligned}$$

27 $$\begin{aligned} 0&= \mathcal{L } u_1, \end{aligned}$$

28 $$\begin{aligned} 0&= \mathcal{L } u_2 + (1-{\overline{u}})\beta _2 [w \otimes u_1]^2 - \beta _1 [w \otimes u_1] {\mathcal{H }} u_1, \end{aligned}$$

29 $$\begin{aligned} \frac{1}{r_c}\frac{\partial u_1}{\partial T}&= \mathcal{L } u_3 + \frac{\delta }{r_c^2} \frac{\partial u_1}{\partial t}+ (1-{\overline{u}}) 2 \beta _2 [w \otimes u_1][w \otimes u_2] + (1-{\overline{u}})\beta _3 [w \otimes u_1]^3 \nonumber \\&-\beta _1 [w \otimes u_1]{\mathcal{H }} u_2 -\beta _1 [w \otimes u_2]{\mathcal{H }} u_1- \beta _0 \int _{t-1}^t \frac{\partial u_1(x,s,T)}{\partial T}(s-t) {{\text{ d }}} s, \nonumber \\ \end{aligned}$$

where

30 $$\begin{aligned} \mathcal{L } = - \frac{1}{r_c} \frac{\partial }{\partial t} -1 +\beta _1 (1-{\overline{u}}) w \otimes - \beta _0 {\mathcal{H }}, \end{aligned}$$

and

31 $$\begin{aligned} {\mathcal{H }} u(x,t,T) = \int _{t-1}^t u(x,s,T) {\text{ d }} s. \end{aligned}$$

Here $\beta _0=f({\overline{u}}),\, \beta _1=f^{\prime }({\overline{u}})=\beta f({\overline{u}})(1-f({\overline{u}})),\, \beta _2=f^{\prime \prime }({\overline{u}})/2=\beta ^2 f({\overline{u}})(1-f({\overline{u}}))(1-2f({\overline{u}}))/2$ and $\beta _3=f^{\prime \prime \prime }({\overline{u}})/6=\beta ^3 f({\overline{u}})(1-f({\overline{u}}))(1-6f({\overline{u}})+6f^2({\overline{u}}))/6$. The first equation (26) fixes the steady state ${\overline{u}}$. The second equation (27) is linear with solutions $u_1=Z_L {\text{ e }}^{i(\omega _c t +k_c x)} + Z_R {\text{ e }}^{i(\omega _c t -k_c x)} + \text{ cc }$, which is equivalent to saying that the kernel of $\mathcal{L }$ is spanned by the functions $\{{}\cos (\omega _c t +k_c x), \cos (\omega _c t -k_c x), \sin (\omega _c t +k_c x), \sin (\omega _c t -k_c x)\}$. A dynamical equation for the complex amplitudes $Z_{L,R}(T)$ (and we do not treat here any slow spatial variation) can be obtained by deriving solvability conditions for the higher-order equations, a method known as the Fredholm alternative. These equations have the general form $\mathcal{L } u_n = v_n(u_0,u_1,\ldots , u_{n-1})$ (with $\mathcal{L }u_1=0$). We define the inner product of two periodic functions (with spatial periodicity $2\pi /k_c$ and temporal periodicity $2 \pi /\omega _c$) as

32 $$\begin{aligned} \langle U, V \rangle = \frac{k_c \omega _c}{(2 \pi )^2}\int _0^{2 \pi /k_c} \int _0^{2 \pi /\omega _c} U^*(x,t) V(x,t) {\text{ d }} x {\text{ d }} t, \end{aligned}$$

where $^*$ denotes complex conjugation. The adjoint of $\mathcal{L }$ with respect to this inner product can be found as

33 $$\begin{aligned} \mathcal{L }^\dag = \frac{1}{r_c} \frac{\partial }{\partial t} -1 +\beta _1 (1-{\overline{u}}) w \otimes - \beta _0 {\mathcal{H }}^\dag , \end{aligned}$$

where

34 $$\begin{aligned} {\mathcal{H }}^\dag u(x,t,T) = \int _t^{t+1} u(x,s,T) {\text{ d }} s . \end{aligned}$$

We observe that the kernel of $\mathcal{L }^\dag$ is spanned by the same set of functions as the kernel of $\mathcal{L }$. Hence the solvability condition takes the form

35 $$\begin{aligned} \langle {\text{ e }}^{\pm i(\omega _c t \mp k_c x)}, \mathcal{L } u_n \rangle = \langle \mathcal{L }^\dag {\text{ e }}^{\pm i(\omega _c t \mp k_c x)}, v_n \rangle = 0, \quad n \ge 2 . \end{aligned}$$

The solvability condition with $n=2$ is automatically satisfied. A comparison of the terms in (28) suggests writing $u_2$ in the form

36 $$\begin{aligned} u_2 = \alpha _0&+\alpha _1 {\text{ e }}^{2 i (k_c x +\omega _c t)} + \alpha _2 {\text{ e }}^{2 i (k_c x -\omega _c t)} + \alpha _3 {\text{ e }}^{-2 i (k_c x +\omega _c t)} +\alpha _4 {\text{ e }}^{-2 i (k_c x -\omega _c t)} \nonumber \\&+ \alpha _5 {\text{ e }}^{2 i k_c x} + \alpha _6 {\text{ e }}^{-2 i k_c x} + \alpha _7 {\text{ e }}^{2 i \omega _c t} + \alpha _8 {\text{ e }}^{-2 i \omega _c t}+ \alpha _9 u_1. \end{aligned}$$

For $n=3$ the calculation of the solvability condition, projecting onto $\phi ={\text{ e }}^{i(\omega _c t + k_c x)}$, is facilitated with the following results

37 $$\begin{aligned}&\left\langle \phi , \frac{\partial u_1}{\partial T} \right\rangle = \frac{\text{ d } Z_L}{\text{ d } T}, \quad \left\langle \phi , \frac{\partial u_1}{\partial t} \right\rangle = i \omega _c Z_L , \nonumber \\&\left\langle \phi , [w \otimes u_1][w \otimes u_2] \right\rangle \!=\! {\widehat{W}}(k_c) [Z_L \alpha _0 \!+\!Z_R^{*} \alpha _7] \!+\!{\widehat{W}}(k_c){\widehat{W}}(2k_c)[Z_L^* \alpha _1 \!+\! Z_R \alpha _5],\nonumber \\&\left\langle \phi ,[w \otimes u_1]^3 \right\rangle = 3{\widehat{W}}(k_c)^3Z_L (|Z_L|^2+2|Z_R|^2), \nonumber \\&\left\langle \phi , [w \otimes u_1]\mathcal{H } u_2 \right\rangle \!=\! {\widehat{W}}(k_c) [Z_L \alpha _0 \!+\!Z_R \alpha _5+Z_L^* \alpha _1 {\widehat{H}}(2 \omega _c) \!+\! Z_R^* \alpha _7 {\widehat{H}}(2 \omega _c)],\qquad \quad \\&\left\langle \phi , [w \otimes u_2]\mathcal{H } u_1 \right\rangle = [Z_L \alpha _0 {\widehat{H}}(\omega _c) +Z_R^* \alpha _7 {\widehat{H}}(-\omega _c)]\nonumber \\&+ {\widehat{W}}(2k_c)[ Z_L^* \alpha _1 {\widehat{H}}(-\omega _c) + Z_R^* \alpha _5 {\widehat{H}}(\omega _c)], \nonumber \\&\left\langle \phi , \int _{t-1}^t \frac{\partial u_1(x,s,T)}{\partial T}(s-t) {\text{ d }} s \right\rangle = \frac{\text{ d } Z_L}{\text{ d } T} \frac{1}{i \omega _c} [1-{\widehat{H}}(\omega _c)(1+i \omega _c)] .\nonumber \end{aligned}$$

Here ${\widehat{H}}(\omega _c)=(1-{\text{ e }}^{-i \omega _c})/(i \omega _c)=[(1-{\overline{u}}) \beta _1 {\widehat{W}}(k_c)-1-i \omega _c/r_c]/\beta _0$ (after using the bifurcation condition $\mathcal{E }(i \omega _c,k_c)=0$). Substitution of (36) into (28) and equating powers of exponentials gives $\alpha _0=a_0 (|Z_L|^2+|Z_R|^2),\, \alpha _1=a_1 Z_L^2,\, \alpha _5=a_5 Z_L Z_R^*$ and $\alpha _7=a_7 Z_L Z_R$ where

38 $$\begin{aligned} a_0&= \frac{-2 (1-{\overline{u}}) \beta _2 {\widehat{W}}(k_c)^2 +\beta _1 {\widehat{W}}(k_c)[{\widehat{H}}(\omega _c)+{\widehat{H}}(-\omega _c)] }{\beta _1 (1-{\overline{u}})-\beta _0-1}, \end{aligned}$$

39 $$\begin{aligned} a_1&= \frac{- (1-{\overline{u}}) \beta _2 {\widehat{W}}(k_c)^2 +\beta _1 {\widehat{W}}(k_c){\widehat{H}}(\omega _c) }{\beta _1 (1-{\overline{u}}) {\widehat{W}}(2 k_c)-\beta _0 {\widehat{H}}(2 \omega _c)-1-2 i \omega _c/r_c} , \end{aligned}$$

40 $$\begin{aligned} a_5&= \frac{-2 (1-{\overline{u}}) \beta _2 {\widehat{W}}(k_c)^2 +\beta _1 {\widehat{W}}(k_c)[{\widehat{H}}(\omega _c)+{\widehat{H}}(-\omega _c)]}{\beta _1 (1-{\overline{u}}) {\widehat{W}}(2 k_c)-\beta _0-1}, \end{aligned}$$

41 $$\begin{aligned} a_7&= \frac{-2 (1-{\overline{u}}) \beta _2 {\widehat{W}}(k_c)^2 +\beta _1 {\widehat{W}}(k_c){\widehat{H}}(\omega _c)}{\beta _{1} (1-{\overline{u}})-\beta _{0} {\widehat{H}}(2 \omega _{c})-1-2 {i} \omega _{c}{/} r_{c}} . \end{aligned}$$

Finally using $\langle \phi , \mathcal{L } u_3 \rangle =0$ and the above results gives Eq. (14) with

42 $$\begin{aligned} {\varLambda }&= i \omega _c \delta /\kappa , \quad \kappa = r_c \left[ 1+ \beta _0 r_c[1-{\widehat{H}}(\omega _c)(1+i \omega _c)]/(i \omega _c) \right] , \end{aligned}$$

43 $$\begin{aligned} {\varPhi }&= r_c^2 \left[ (1-\overline{u}) 2 \beta _2 {\widehat{W}}(k_c)(a_0+{\widehat{W}}(2k_c)a_1) + (1-\overline{u})3\beta _3{\widehat{W}}^3(k_c) \right. \nonumber \\&\left. - \beta _1 \left( ({\widehat{W}}(k_c)+{\widehat{H}}(\omega _c))a_0+({\widehat{W}}(k_c){\widehat{H}}(2 \omega _c)+{\widehat{W}}(2k_c){\widehat{H}}(-\omega _c)) a_1\right) \right] /\kappa , \nonumber \\ \end{aligned}$$

44 $$\begin{aligned} {\varPsi }&= r_c^2 \left[ (1-\overline{u}) 2 \beta _2 {\widehat{W}}(k_c)(a_0+a_7+{\widehat{W}}(2k_c)a_5) + (1-\overline{u})6\beta _3{\widehat{W}}^3(k_c) \right. \nonumber \\&- \beta _1\left( \left( {\widehat{W}}(k_c)+{\widehat{H}}(\omega _c)\right) a_0+\left( {\widehat{W}}(k_c) +{\widehat{W}}(2k_c){\widehat{H}}(\omega _c)\right) a_5\right. \nonumber \\&\left. \left. +\left( {\widehat{W}}(k_c){\widehat{H}}(2\omega _c)+{\widehat{H}}(-\omega _c)\right) a_7\right) \right] / \kappa . \end{aligned}$$

A similar analysis, projecting onto ${\text{ e }}^{i(\omega _c t -k_c x)}$, yields (15).

## References

[CR1] Beurle RL (1956) Properties of a mass of cells capable of regenerating pulses. Philos Trans R Soc Lond B 240:55–94

[CR2] Bressloff PC (2012). Spatiotemporal dynamics of continuum neural fields. J Phys A.

[CR3] Coombes S (2013). Neural field theory, chap. Tutorial on neural field theory.

[CR4] Coombes S (2003). Waves and bumps in neuronal networks with axo-dendritic synaptic interactions. Phys D.

[CR5] Curtu R, Ermentrout B (2001). Oscillations in a refractory neural net. J Math Biol.

[CR6] Curtu R, Ermentrout B (2004). Pattern formation in a network of excitatory and inhibitory cells with adaptation. SIAM J Appl Dyn Syst.

[CR7] Ermentrout GB (1998). Neural nets as spatio-temporal pattern forming systems. Rep Prog Phys.

[CR8] Ermentrout GB, McLeod JB (1993). Existence and uniqueness of travelling waves for a neural network. Proc R Soc Edinb A.

[CR9] Evans JW (1982). Double impulse solutions in nerve axon equations. SIAM J Appl Math.

[CR10] Feroe JA (1982). Existence and stability of multiple impulse solutions of a nerve equation. SIAM J Appl Math.

[CR11] Gonchenko SV (1997). Complexity in the bifurcation structure of homoclinic loops to a saddle-focus. Nonlinearity.

[CR12] Hastings SP (1982). Single and multiple pulse waves for the FitzHugh-Nagumo equations. SIAM J Appl Math.

[CR13] Hoyle R (2006). Pattern formation: an introduction to methods.

[CR14] Huang X (2004). Spiral waves in disinhibited mammalian neocortex. J Neurosci.

[CR15] Keener J, Sneyd J (1998). Mathematical physiology.

[CR16] Kilpatrick ZP, Bressloff PC (2010). Spatially structured oscillations in a two-dimensional neuronal network with synaptic depression. J Comput Neurosci.

[CR17] Kuznetsov YA (1994). Impulses of a complicated form in models of nerve conduction. Selecta Mathematica (formerly Sovietica).

[CR18] Lin XB (1990). Using Melnikov’s method to solve Silnikov’s problems. Proc R Soc Edinb A.

[CR19] Lord GJ, Coombes S (2002). Traveling waves in the Baer and Rinzel model of spine studded dendritic tissue. Phys D.

[CR20] Meijer HGE, Meyers RA (2009). Numerical bifurcation analysis. Encyclopedia of complexity and systems science.

[CR21] Miller RN, Rinzel J (1981). The dependence of impulse propagation speed on firing frequency, dispersion, for the Hodgkin-Huxley model. Biophys J.

[CR22] Pinto DJ, Ermentrout GB (2001) Spatially structured activity in synaptically coupled neuronal networks: I. Travelling fronts and pulses. SIAM J Appl Math 62:206–225

[CR23] Rinzel J, Maginu K, Vidal C, Pacault A (1984). Kinematic analysis of wave pattern formation in excitable media. Non-equilibrium dynamics in chemical systems.

[CR24] Röder G (2007). Wave trains in an excitable FitzHugh-Nagumo model: bistable dispersion relation and formation of isolas. Phys Rev E.

[CR25] Roose D, Szalai R (2007) Continuation methods for dynamical systems: path following and boundary value problems. Continuation and bifurcation analysis of delay differential equations, Springer-Canopus, Verlag, pp 359–399

[CR26] Taylor PN, Baier G (2011). A spatially extended model for macroscopic spike-wave discharges. J Comput Neurosci.

[CR27] Venkov NA (2007). Dynamic instabilities in scalar neural field equations with space-dependent delays. Phys D.

[CR28] Wilson HR, Cowan JD (1972). Excitatory and inhibitory interactions in localized populations of model neurons. Biophys J.

[CR29] Wilson HR, Cowan JD (1973) A mathematical theory of the functional dynamics of cortical and thalamic nervous tissue. Kybernetik 13:55–80 10.1007/BF00288786 4767470

